# Cerebrospinal fluid levels of GFAP and pNF-H are elevated in patients with chronic spinal cord injury and neurological deterioration

**DOI:** 10.1007/s00701-020-04422-6

**Published:** 2020-06-25

**Authors:** Ulrika Holmström, Parmenion P. Tsitsopoulos, Anders Holtz, Konstantin Salci, Gerry Shaw, Stefania Mondello, Niklas Marklund

**Affiliations:** 1 Department of Neuroscience, Neurosurgery, Uppsala University, Uppsala, Sweden; 2 Department of Neurosurgery, Hippokratio General Hospital, Aristotle University Faculty of Medicine,, Thessaloniki, Greece; 3 Department of Neuroscience, College of Medicine, University of Florida, Gainesville, FL USA; 4 Department of Biomedical and Dental Sciences and Morphofunctional Imaging, University of Messina, Messina, Italy; 5 Department of Clinical Sciences Lund, Neurosurgery Lund University, Skåne University Hospital, Lund, Sweden

**Keywords:** Spinal cord injury, Biomarkers, Syringomyelia, Cerebrospinal fluid, Tethering of the spinal cord (TSC), Glial fibrillary acidic protein (GFAP), Ubiquitin C-terminal hydrolase L1 (UCH-L1), Neurofilament, Post-traumatic myelopathy

## Abstract

**Background:**

Years after a traumatic spinal cord injury (SCI), a subset of patients may develop progressive clinical deterioration due to intradural scar formation and spinal cord tethering, with or without an associated syringomyelia. Meningitis, intradural hemorrhages, or intradural tumor surgery may also trigger glial scar formation and spinal cord tethering, leading to neurological worsening. Surgery is the treatment of choice in these chronic SCI patients.

**Objective:**

We hypothesized that cerebrospinal fluid (CSF) and plasma biomarkers could track ongoing neuronal loss and scar formation in patients with spinal cord tethering and are associated with clinical symptoms.

**Methods:**

We prospectively enrolled 12 patients with spinal cord tethering and measured glial fibrillary acidic protein (GFAP), ubiquitin C-terminal hydrolase L1 (UCH-L1), and phosphorylated Neurofilament-heavy (pNF-H) in CSF and blood. Seven patients with benign lumbar intradural tumors and 7 patients with cervical radiculopathy without spinal cord involvement served as controls.

**Results:**

All evaluated biomarker levels were markedly higher in CSF than in plasma, without any correlation between the two compartments. When compared with radiculopathy controls, CSF GFAP and pNF-H levels were higher in patients with spinal cord tethering (*p* ≤ 0.05). In contrast, CSF UCH-L1 levels were not altered in chronic SCI patients when compared with either control groups.

**Conclusions:**

The present findings suggest that in patients with spinal cord tethering, CSF GFAP and pNF-H levels might reflect ongoing scar formation and neuronal injury potentially responsible for progressive neurological deterioration.

## Introduction

Glial scar formation, resulting in spinal cord tethering, may become clinically evident many years following spinal cord injury (SCI), irrespective of the initial injury mechanism or severity [11, 12, 14, 24]. When the spinal cord is tethered to the surrounding dura, impaired cord pulsations, reduced flow of the cerebrospinal fluid, and/or spinal cord traction can lead to neurological dysfunction and/or spinal cord cyst formation (syringomyelia) [18, 33, 52]. Most symptomatic spinal cord tethering occurs secondary to traumatic SCI where up to 5% of patients develop progressive neurological deterioration after a few months up to many years following the initial injury [11, 14, 24, 43]. This clinical entity is named post-traumatic myelopathy. Non-traumatic causes of glial scar formation include infectious or hemorrhagic causes, or may occur in a delayed fashion following intradural surgery [16, 22].

The pathogenesis of progressive glial scarring and how this is associated with neurological dysfunction is incompletely understood. Blood products and factors released from the injured spinal cord at time of initial injury may lead to arachnoiditis and chronic neuroinflammation, which, in turn, causes gradual scarring and tethering of the cord to the surrounding dura mater which may result in progressive neurodegeneration [4, 32, 43, 44, 53]. Animal experiments have shown that spinal cord tethering is an important contributing factor for the formation of intramedullary cysts and progressive spinal cord dysfunction [9, 28]. Treatment of symptomatic spinal cord tethering, regardless of its cause, is surgical [24, 27, 45].

Blood-based markers indicative of glial and neuronal injury have attracted attention over the past decades [37, 39]. Although previous biomarker studies following acute SCI exist [10, 29, 30, 56], data are lacking on spinal cord tethering [2]. Easily accessible, objective, and inexpensive biochemical markers reflecting glial scar formation and progressive neuronal damage are needed and might help to improve diagnosis, monitor ongoing pathophysiological mechanisms, and predict outcome of post-traumatic myelopathy. Such biomarkers could also aid in medical decision-making and permit the development of therapeutic interventions aiming to prevent or optimize surgery of chronic SCI patients.

In the present study, we hypothesized that biomarkers of neuronal/axonal/glial damage would reflect the ongoing pathophysiology and symptoms in patients with spinal cord tethering and, thereby, represent a useful approach to objectively assess the severity of this condition. With these aims in mind, we sampled blood and CSF from patients with spinal cord tethering and examined concentrations of a panel of pathobiologically diverse markers, namely glial fibrillary acidic protein (GFAP), ubiquitin C-terminal hydrolase L1 (UCH-L1) and phosphorylated neurofilament-heavy (pNF-H).

## Materials and methods

### Patients and setting

Enrolled participants represented a mixed cohort of consecutive patients presenting with gradual neurological worsening caused by scar formation/spinal cord tethering following a primary spinal cord injury, as a result of traumatic, infectious, postoperative, or vascular causes (tethered spinal cord—TSC). Congenital spinal cord tethering was an exclusion criterion. All recruited patients presented with clinical (progressive deterioration of motor and/or sensory function) and radiological (spinal cord tethering and/or edema, with or without syringomyelia) features consistent with the diagnosis of tethered spinal cord and were scheduled for microneurosurgical untethering, syringosubarachnoid shunting of associated syringomyelia (when considered necessary), and duraplasty at the Department of Neurosurgery, Uppsala University Hospital, Uppsala, Sweden, between March 2013 and October 2015.

Clinical evaluations were performed preoperatively and at 3 months after surgery by a trained researcher (UH). Functional impairment was assessed using the American Spinal Cord Injury Association (ASIA) Impairment Scale (AIS) [26]. Additional outcome measures included the JOA Cervical Myelopathy Evaluation Questionnaire (JOACMEQ), which evaluates cervical spine function and quality of life [15] and the EQ-5D, a standardized instrument of health status for clinical and economic appraisal [13, 40]. The dimensions of EQ-5D comprise mobility, self-care, usual activities, pain/discomfort, and anxiety/depression and are rated by the responder as 1, no problem; 2, some problems; or 3, extreme problems. This assessment is accompanied by an EQ-VAS value between 0 and 100, rating the actual Quality of Life-situation, where 0 is “worst imaginable health state” and 100 is “best imaginable health state [13, 40].

Postoperative follow-up of the questionnaires was conducted through phone calls by the first author (UH).

### Control patients

A control group of patients undergoing surgery for benign intradural lumbar tumors, without spinal cord involvement and without radiological or clinical signs of tethering, was recruited (Ctrl-T). All tumors were located below the conus medullaris. The surgical approach to the intradural compartment was similar while perioperative CSF and plasma sampling was identical to that used for the tethered spinal cord patients (see below).

Since expression by astrocytic and neuronal markers may be present in some benign intradural tumors, we also recruited a second cohort of consecutive controls comprised of patients with cervical radiculopathy caused by herniated discs or degenerative root canal stenosis (Ctrl-R). All patients underwent a preoperative magnetic resonance imaging (MRI). None had any clinical or radiological evidence of spinal cord compression and myelopathy, or spinal cord signal changes. All patients were planned for decompressive cervical surgery through anterior discectomy and fusion.

### Surgical procedures

**Fig. 1:**
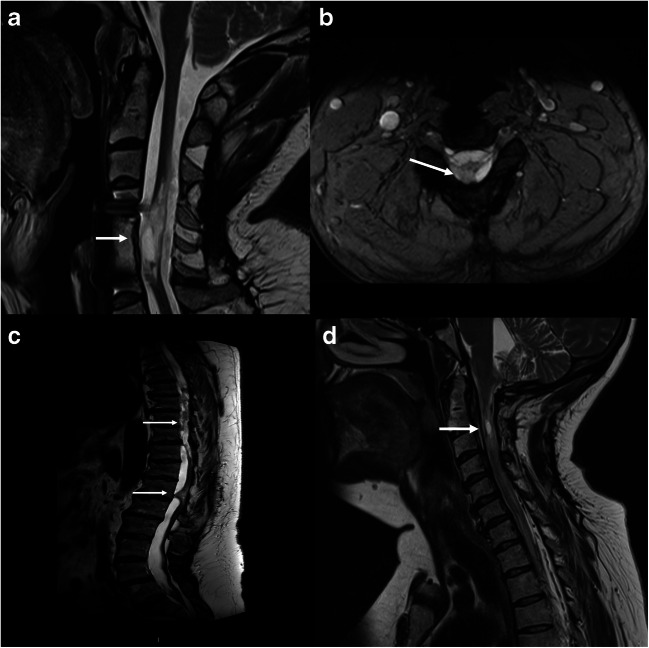
Preoperative magnetic resonance imaging (MRI) of three patients included in the present cohort. **a** Evidence of myelomalacia with spinal cord tethering over 10 years following a traumatic spinal injury, sagittal image (arrow). **b** Spinal cord tethering at C5, axial image. The patient was treated surgically with laminectomy, untethering of the spinal cord and duraplasty (arrow). **c** Patient sustaining an intradural hemorrhage from a routine spinal tap that resulted in neurological deficits and emergency surgical evacuation. At 12 months following the hemorrhage, a gradual yet marked neurological deterioration of lower limb sensory and motor function was observed which was treated by laminectomy, spinal cord untethering, and duraplasty. Note the marked adhesions and deformation of the spinal cord (arrows). **d** Intradural scarring, an intramedullary cyst (arrow), and spinal cord signal changes were found in a patient with previous meningitis and gradual neurological deterioration who was surgically treated by intradural exploration and release of scar tissue. This patient had the highest CSF GFAP levels in the current cohort

**Fig. 2:**
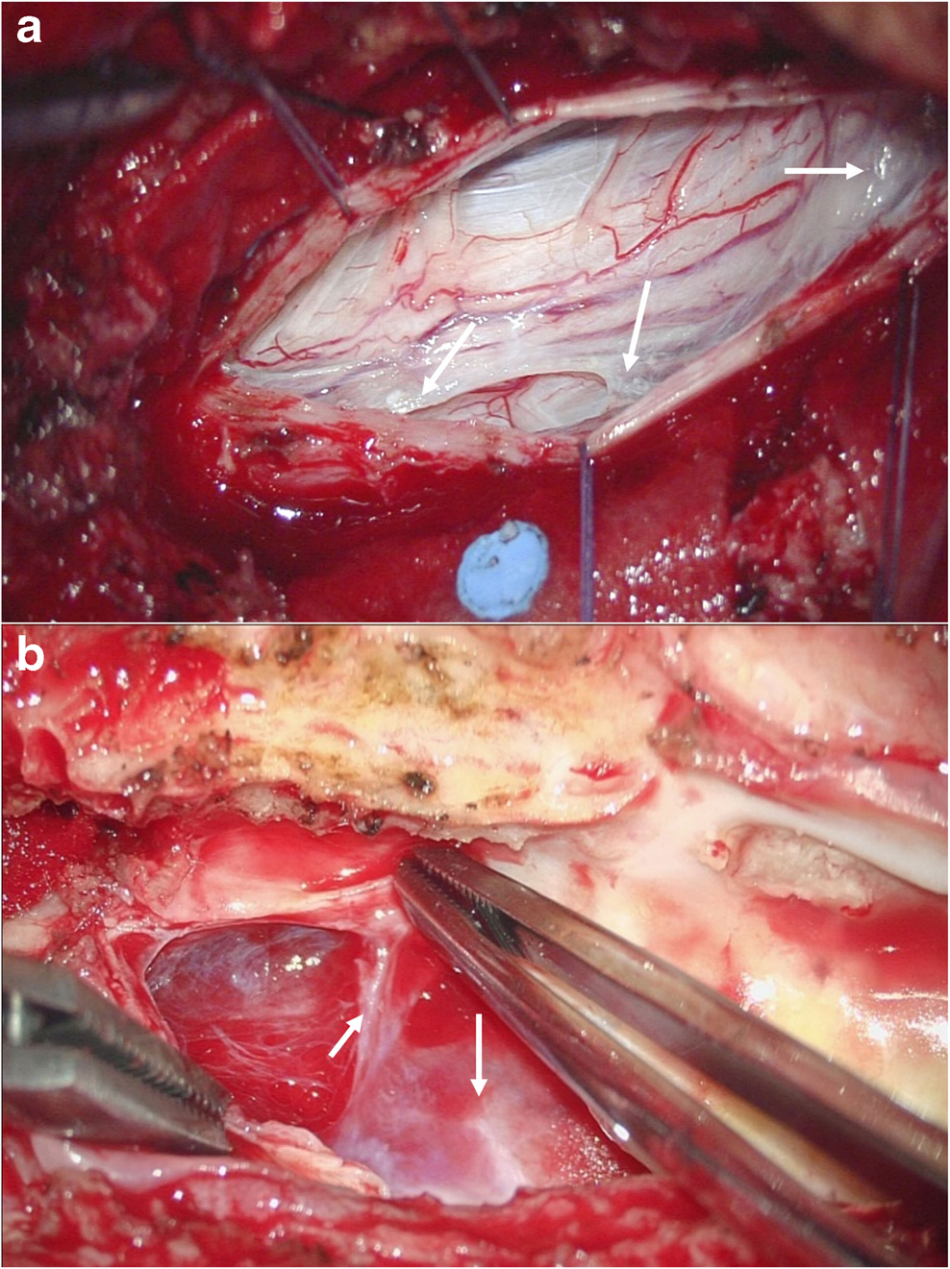
Two intraoperative images showing examples of intradural scar formation with spinal cord tethering to the surrounding dura. **a** A rather mild scar formation was observed (arrows). **b** A thickened arachnoid with extensive scarring was observed (arrow) in a patient with previous intradural spinal hemorrhage

Patient features, spinal configuration, and extent of pathology guided the surgical treatment strategy. All patients had a preoperative MRI (Fig. 1), including axial and sagittal images, for the evaluation of spinal cord pathology including tethering, edema, cysts/syringomyelia, and cord compression. In cases with incomplete spinal cord injury, intraoperative neurophysiological spinal cord monitoring including motor (MEP) and sensory (SEP) evoked potentials was commonly used. The surgical techniques encompassed a limited laminectomy targeted to the intradural pathology, dural opening using microinstruments under microscopic view, and sharp dissection of the scar tissue for the release of the tethered spinal cord (Fig. 2a, b) to restore CSF flow. When an intramedullary cyst persisted after dural opening, as controlled by intraoperative ultrasound, a syringosubarachnoidal shunt was placed [24]. In all patients, the dura was closed and expanded using a dural graft (Durepair®, Medtronic, Memphis, TN) that was sutured using resorbable sutures. All surgeries were performed by a single surgeon (NM).

### Blood and CSF sampling

In patients with spinal cord scar formation undergoing surgical treatment as well as in the tumor control patients, CSF samples for biomarker analyses were obtained during surgery. Prior to dural opening, meticulous hemostasis was achieved to ensure that the surgical area was clear from blood. After the dura was opened by a midline durotomy under the microscope and CSF was released, 3–5 mL of CSF were immediately collected using a syringe attached to a blunt needle. Simultaneously, blood samples were drawn from the same individual.

In the cervical radiculopathy controls, CSF was obtained the day prior to cervical surgery using a routine lumbar spinal tap. Blood samples were also acquired.

CSF and plasma were centrifuged at 3600 rpm at 4 °C for 10 min, aliquoted into cryovials, and stored at − 70 °C until analysis. All procedures were performed according to a standardized protocol and in line with international consensus recommendations [36]. Samples were shipped on dry ice to EnCor Biotechnology (Gainesville, FL, USA) for biomarker analyses.

### Analysis of GFAP, UCH-L1, and pNF-H

CSF and plasma samples were assayed for the presence of pNF-H and UCH-L1 using assays based on previously published ELISAs [23]. The basic assays were modified to run on the MesoScale Discovery (MSD) platform (Rockville, MD), an electrochemiluminescence-based assay in which signal is detected by light emission from a suitably tagged detection reagent. In brief, purified MCA-NAP4 pNF-H capture antibody (EnCor Biotechnology, Gainesville, FL) was used along with affinity purified chicken polyclonal pNF-H detection antibody. The chicken antibody was affinity purified from a commercially available IgY preparation (CPCA-NF-H, EnCor) and was directly labeled with the MSD sulfotag reagent [7, 47]. MCA-NAP4 and the chicken pNF-H antibody were originally raised against a preparation of purified pig pNF-H as previously described [21]. Both MCA-NAP4 and the chicken pNF-H antibody specifically recognize only the phosphorylated axonal form of NF-H protein, and both antibodies bind only to axonal profiles on neurons in tissue culture and in brain sections [7]. For the UCH-L1 assay capture reagent, we used a mouse monoclonal antibody MCA-BH7 (EnCor) as previously described [35]. This was detected with sulfotagged affinity purified rabbit anti-UCH-L1 derived from RPCA-UCH-L1 serum (EnCor). Both antibodies were raised against recombinant full-length human UCH-L1 expressed in and purified from *Escherichia coli*. All antibodies were manufactured and all assays were run in the EnCor Biotechnology laboratory. The pNF-H assay has a lower limit of quantitation (LLOQ) of 5 pg/mL and the UCH-L1 assay has an LLOQ of 15 pg/mL. In both cases, blood samples were diluted 1:2 in 1% bovine serum albumin and 2% Tween 20 in Tris-buffered saline, and 30-μL samples were run in duplicate on standard MSD plates. Incubations with either samples or detection reagent lasted 1 h at room temperature with vigorous shaking, and plates were extensively washed between incubations and prior to signal detection.

GFAP levels were measured with a novel assay which used mouse monoclonal to GFAP MCA-2A5 (EnCor) as the capture reagent. This antibody was originally made against native GFAP purified from pig spinal cord and shows strong binding for human, cow, and pig GFAP in ELISA, Western blotting, and on astrocytic cells but reduced binding to rodent GFAP. As a result, this assay may be less than optimal for studies involving rodents. The purified antibody was applied to MSD plates at 1 μg/mL in PBS overnight at 4 °C. The plates were blocked as above and the samples added in dilution buffer. The detection antibody was affinity purified from RPCA-GFAP (EnCor), a rabbit serum generated using full-length recombinant human GFAP isotype 1 as the immunogen. This recombinant protein (Prot-r-GFAP, EnCor) was also used as the protein standard. The purified rabbit GFAP antibody was reacted directly with the sulfotag reagent as described above. The assay detected human GFAP with an LLOQ of 10 pg/mL.

### Statistical analysis

Exploratory analysis was carried out to determine the distribution of the demographic and clinical variables. Subject characteristics were summarized using standard descriptive statistics. Continuous variables were described as mean (SD) or median (IQR), as appropriate, and categorical data were summarized as absolute frequencies and percentages. Since biomarker levels did not meet the criteria for normal distribution, non-parametric statistics were used. The Kruskal-Wallis test was used for group-wise comparisons, followed by, if significant, pair-wise comparisons using the Mann-Whitney *U* test. Correlations between biomarkers and their relation to radiological parameters were analyzed using the Spearman rank correlation test. All conducted hypotheses tests were two-tailed and a *p* value ≤ 0.05 was considered significant. The statistical analyses were performed using GraphPad Prism 7 for Mac and Windows (San Diego, CA, USA).

## Results

### Patient description and control population

Thirteen consecutive patients were initially included, but one patient was excluded because of a co-existing motor neuron disease. The average age was 50 ± 14 years, 4 of 12 (33%) subjects were female, and the median AIS grade was C (Table 1). With the exception of one patient who had an initial subarachnoid hemorrhage from a ruptured posterior inferior cerebellar artery (PICA) aneurysm, all patients had prior spinal surgery at time of initial spinal injury. Six (50%) participants had a previous traumatic spinal cord injury (SCI), of whom four were motor incomplete (AIS C-D) and two were motor complete (AIS A-B) SCIs. The symptom duration ranged from 1 month up to more than 2 years prior to surgery. There were no surgical complications (infection, wound healing problems, postoperative hemorrhages) except for symptomatic re-tethering in three patients (Table 1).

**Table 1:** Baseline characteristics of the included patients operated for symptomatic spinal cord tethering. All patients except for #12 had previous spinal surgery.

Patient number	Age (y)	Gender	Underlying pathology	Symptom duration (m)	AIS Grade	Spinal Level
1^*^	62	M	Trauma	1	AIS-C	C4
2	53	M	Trauma	8	AIS-D	Th6
3	66	M	Trauma	6	AIS-C	Th7
4^*^	50	M	Trauma	12	AIS-A	C4
5	46	M	Previous surgery for intramedullary tumour	10	AIS-C	C4
6	51	F	Trauma	12	AIS-D	C3
7	22	F	Dermoid cyst surgery at childhood	10	AIS-C	T3
8	67	F	Intradural hemorrhage	6	AIS-D	T4
9^*^	62	M	Intradural hemorrhage	12	AIS-C	T5
10^†^	46	F	Neonatal meningitis	>24	AIS-B	C0-C3
11	30	M	Trauma	24	AIS-A	C3
12	46	M	SAH with intradural hemorrhage	9	AIS-D	T7

*AIS* American Spinal Cord Injury Association Injury Scale, *C* Cervical, *F* Female, *L* Lumbar, *M* Male, *m* Months, *SAH* Subarachnoid hemorrhage, *T* Thoracic, *y* Years. * Patients who were reoperated due to re-tethering, ^†^ Subject with the highest CSF GFAP levels

**Fig. 3:**
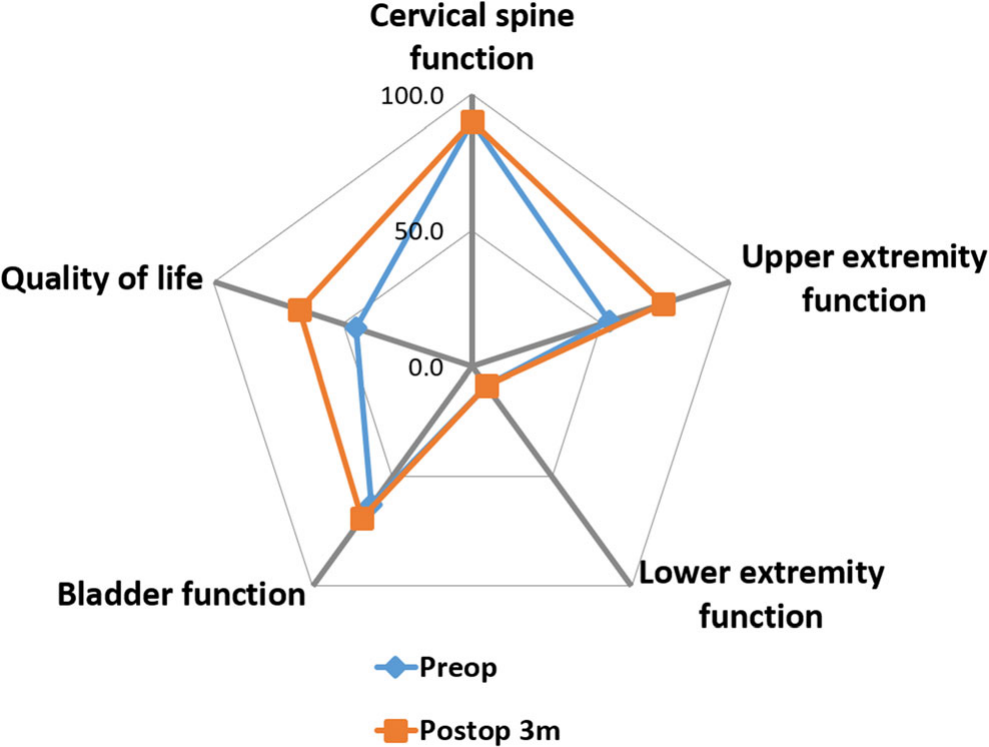
Outcome was assessed using the Japanese Orthopedic Association (JOA) Cervical Myelopathy Evaluation Questionnaire (JOACMEQ) prior to surgery and at 3 months post-surgery in 12 patients on 15 occasions. As expected, no changes in the neurological level and function were found but upper extremity function and quality of life aspects significantly improved following surgery (*p* < 0.05)

The five EQ-5D domains for the group regarding mobility, self-care, usual activities, pain, and anxiety/depression were not altered by surgery. EQ-5D-VAS self-assessed health status significantly improved following surgery (42.7 ± 23.4 vs. 64.3 ± 13.2; *p* = 0.001). The JOACMEQ, evaluated before and after surgery, showed significantly higher values for quality of life (QoL) and upper extremity function postoperatively (*p* < 0.05; Fig. 3).

The tumor control group (Ctrl-T) consisted of eight patients surgically treated for lumbar benign tumors in whom both CSF and blood samples were obtained (Table 2). One patient with a ganglioglioma was found to express intense GFAP staining on histology and was eventually excluded from analysis. Thus, seven tumor controls were analyzed (mean age 56.9 ± 16 years; four female and three male). There were no complications in this cohort of patients. The pathoanatomical diagnosis (PAD) is shown in Table 2.

**Table 2:** Control patients with lumbar intradural tumors (Ctrl-T) or with cervical radiculopathy (Ctrl-R) from whom cerebrospinal fluid was obtained at the time of initial dural opening (see text for details)

	Patient Number	Age (y)	Gender	Underlying pathology	Spinal Level
Ctrl-T	1	61	M	Schwannoma Grade 1 *	L1-2
	2	31	F	Ependymoma Grade 2	L1-2
	3	45	M	Schwannoma Grade 1	L2
	4	69	F	Schwannoma Grade 1	L2-3
	5	51	F	Schwannoma Grade 1	L3-4
	6	59	F	Paraganglioma Grade 1	L3
	7	80	M	Schwannoma Grade 1	L2
Ctrl-R	1	45	M	Cervical radiculopathy	C6-C7
	2	49	F	Cervical radiculopathy	C5-C6
	3	50	F	Cervical radiculopathy	C5-C6
	4	52	M	Cervical radiculopathy	C3-C4, C5-C6
	5	44	M	Cervical radiculopathy	C6-C7
	6	39	M	Cervical radiculopathy	C5-C6
	7	37	F	Cervical radiculopathy	C6-7

*C* cervical, *F* Female, *L* Lumbar, *M* Male, *Y Years*

*Grade according to the 2007 World Health Organization grading system of CNS tumors

The other control group included 7 patients with cervical radiculopathy (Ctrl-R; three female and four male, mean age 45.1 ± 6 years; Table 2). All underwent uneventful anterior cervical discectomy and fusion.

### CSF and plasma biomarkers

#### GFAP levels (Fig. 4a, d)

**Fig. 4:**
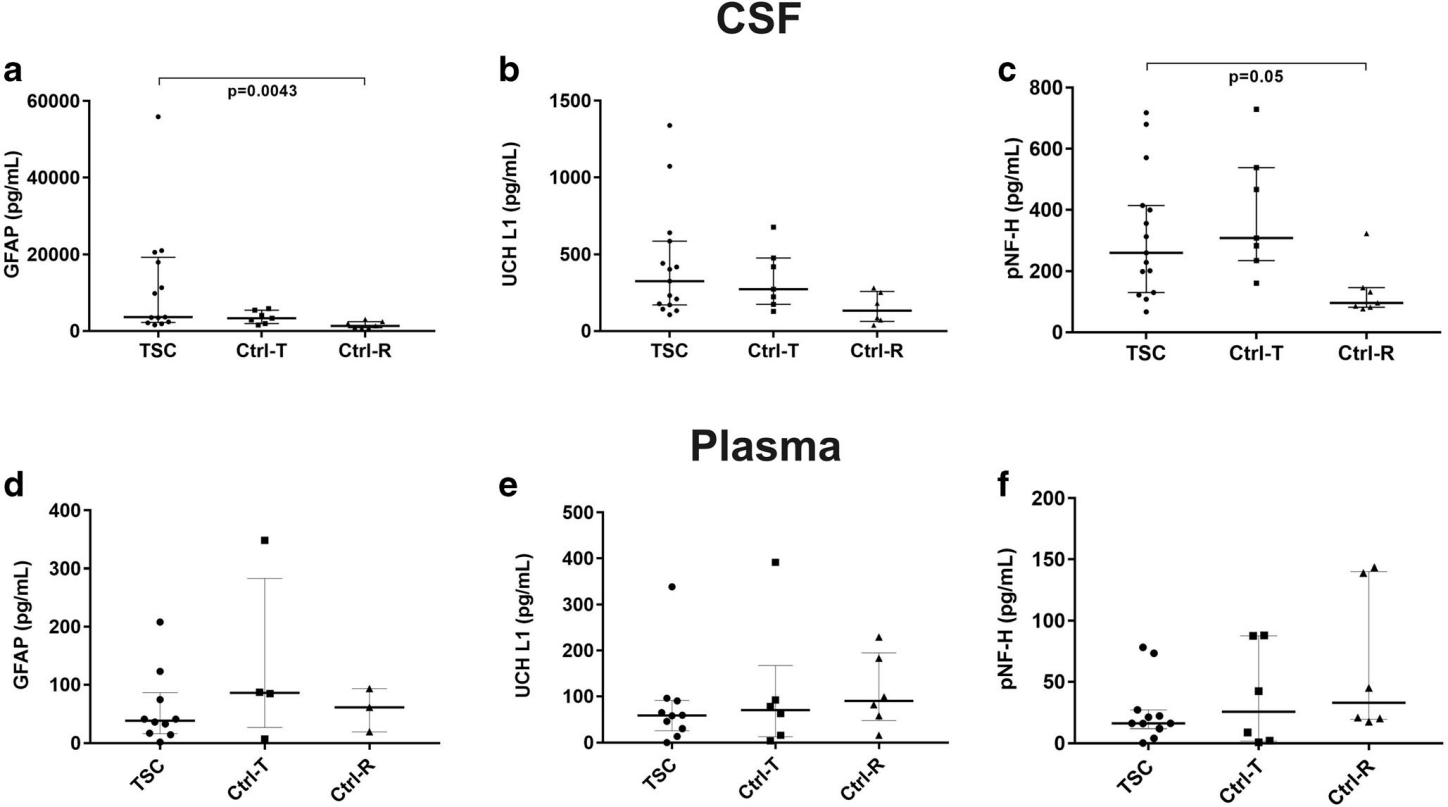
Plasma and cerebrospinal fluid (CSF) biomarker levels in patients undergoing surgery due to neurological deterioration caused by intradural scar formation. Control groups comprised of patients operated on for intradural, lumbar tumors (Ctrl-T), and patients with cervical radiculopathy without spinal cord involvement selected for cervical decompression surgery by anterior discectomy and fusion (Ctrl-R). Data is presented for glial fibrillary acidic protein (GFAP), ubiquitin C-terminal hydrolase L1 (UCH-L1), and phosphorylated neurofilament-heavy (pNF-H). Data is presented as medians, 25th and 75th percentile, and individual values. **a**–**c** Cerebrospinal fluid (CSF) biomarkers. In the TSC and Ctrl-T groups, CSF biomarkers were sampled intraoperatively whereas in patients with cervical radiculopathy (Ctrl-R), the samples were obtained preoperatively via a routine spinal tap. Compared to patients with cervical radiculopathy, the levels of GFAP and pNF-H although not UCH-L1 were higher in those with chronic spinal cord injury/spinal cord tethering and intradural scar formation. **d**–**f** Plasma biomarkers. In patients with chronic spinal cord injury/tethered spinal cord, the biomarker levels in plasma were similar to those with intradural lumbar tumors or cervical radiculopathy control groups for all evaluated biomarkers

CSF levels of GFAP were significantly higher in the tethered spinal cord (TSC) group (median 3605, range 1575–55,909 pg/mL) than in the Ctrl-R (median 1304, range 778–3014 pg/mL; *p* < 0.05), but did not differ from those of the Ctrl-T group (median 3301, range 1565–5828 pg/mL) (Fig. 4a). Plasma levels of GFAP did not differ between the TSC (median 38.3, range 1.7–208.2 pg/mL), Ctrl-T (median 80.8, range 7.2–348.6 pg/mL), and Ctrl-R (median 73.8, range 19.6–93.5 pg/mL) groups (Fig. 4d).

#### UCH-L1 levels (Fig. 4b, e)

UCH-L1 levels in CSF did not differ significantly between the TSC (median 325.7, range 106.3–1340 pg/mL), Ctrl-T (median 272.9, range 128.1–676.1 pg/mL), and Ctrl-R (median 133.6, range 37.9–280.1 pg/mL) groups, though there was a trend towards increased levels in TSC (Fig. 4b). Like GFAP, UCH-L1 levels in plasma were similar in the TSC (median 58.4, range 0.02–338.8 pg/mL), Ctrl-T (median 70.3, range 3.9–391.5 pg/mL), and Ctrl-R (median 73.0, range 12.1–229.5 pg/mL) groups (Fig. 4e).

#### pNF-H levels (Fig. 4c,f)

CSF levels of pNF-H were significantly higher in the TSC group (median 260.1, range 66.9–718.2 pg/mL) than in the Ctrl-R group (median 96.3, range 77.3–322.1 pg/mL; *p* < 0.05), but did not differ significantly from those in the Ctrl-T group (median 307.9, range 160.9–729.4 pg/mL; Fig. 4c). Plasma pNF-H levels did not differ between the TSC (median 16.2, range 0.2–78.3 pg/mL), Ctrl-T (median 8.7, range 0.8–88.1 pg/mL), and Ctrl-R (median 20.8, range 0.2–143.5 pg/mL) groups (Fig. 4f).

### Complications

Three patients were re-operated (Table 1). Patient no. 1, an incomplete SCI patient (AIS C), experienced worsening in gait function and was re-operated due to spinal cord tethering 17 months after the initial surgery. He showed marked improvement after the second surgery. CSF biomarkers were similar in both surgeries.

In patient no. 4, a C4 AIS A post-traumatic patient, CSF UCH-L1 and pNF-H levels were 584.9 and 570.8 pg/mL, respectively, at the initial surgery. At the second surgery (performed 18 months after the initial operation due to progressive worsening of wrist extension function), both GFAP and pNF-H levels in CSF were markedly increased (9847.0 and 718.2 pg/mL, respectively). UCH-L1 levels were similar to the other patients in the cohort (231.7 pg/mL) and substantially lower compared with the first surgery.

Patient no. 9 had an unfortunate intradural hemorrhage secondary to spinal anesthesia and later developed severe spinal cord tethering in the thoracolumbar region in combination with neurological deterioration (Fig. 1c). At the initial surgery, CSF GFAP levels were high (11323.1 pg/mL), although UCH-L1 and pNF-H levels were not (170.7 and 399.7 pg/mL, respectively). At 4 months postoperatively, no improvement was noted and on MRI evidence of re-tethering was found. Biomarker levels were lower compared with the initial surgery (GFAP 2121.4 pg/mL, UCH-L1 133.7 pg/mL and pNF-H 108.8 pg/mL).

#### Illustrative case

A 45-year-old female patient had suffered neonatal meningitis and was later treated with a ventriculoperitoneal shunt that was revised on numerous occasions. She then experienced a progressive tetraparesis and became unable to walk. She underwent surgical decompression at C1–C3 on two occasions, 4 and 6 years previously, without improvement. Due to continuous deterioration of upper extremity function, an MRI was performed (Fig. 1d) that showed a C0–C2 cyst and signs of intradural tethering. A C4 decompression and intradural exploration of C0–C3 was performed. At surgery, dense adhesions were found in addition to a markedly thickened arachnoid membrane. In the CSF obtained at dural opening, exceedingly high GFAP levels (55909 pg/mL) were observed. UCH-L1 levels (1074 pg/mL) were the second highest in this cohort, whereas the pNF-H levels were on an average level (355.5 pg/mL; Fig. 4). Blood biomarker levels were all within the normal range. Postoperatively, initial improvement in hand function was observed.

### Correlations between biomarker levels and clinical factors

**Fig. 5:**
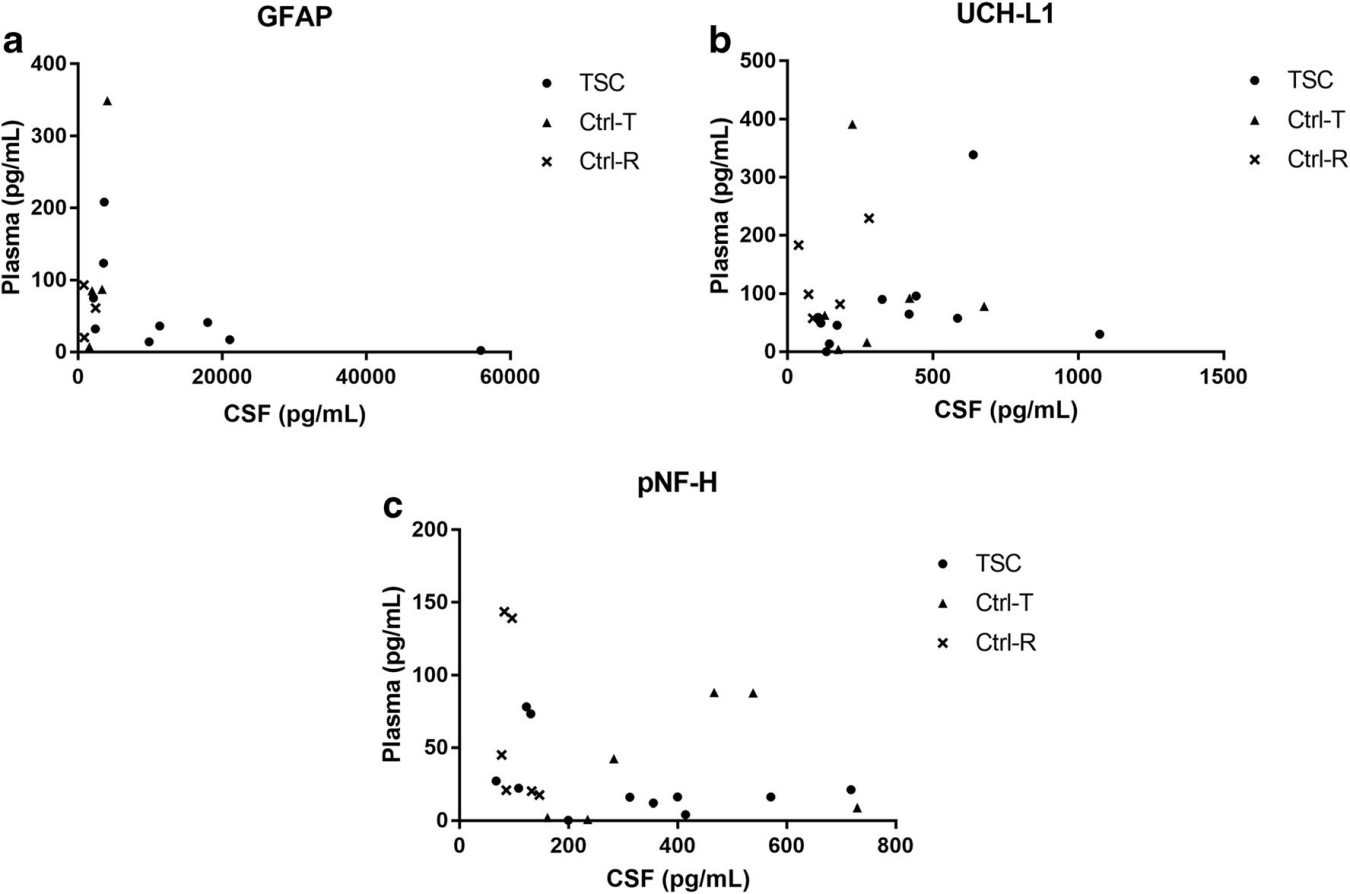
Correlations between plasma and cerebrospinal (CSF) levels of glial fibrillary acidic protein (GFAP), ubiquitin C-terminal hydrolase L1 (UCH-L1), and phosphorylated neurofilament-heavy (pNF-H) (Spearman’s rank correlation). The analyzed groups were patients with neurological deterioration and tethering of the spinal cord (TSC), controls with intradural lumbar tumors without spinal cord involvement (Ctrl-T), and controls with cervical disc disorders causing cervical radiculopathy without spinal cord involvement (Ctrl-R). There were large differences between CSF and plasma levels but no significant correlations between the compartments for any of the analyzed groups were found

CSF and blood biomarker levels did not correlate with age, symptom duration, injury level, and disease severity as assessed by AIS grade, EQ-5D, and JOACMEQ before surgery. As expected, biomarker levels were higher in CSF than in plasma, but no correlation between the two compartments for any of the evaluated biomarkers was found (*p* > 0.05; Fig. 5).

## Discussion

In this study, the first to evaluate CSF and blood biomarkers in patients with spinal cord tethering and neurological deterioration, we observed that CSF concentrations of GFAP and pNF-H are increased in tethered cord SCI patients compared with radiculopathy controls without evidence of spinal cord compression. These elevated concentrations of biomarkers of neuronal/axonal injury and glial scar formation may reflect ongoing processes of clinical relevance. Studies on a larger patient population may provide insight into the mechanisms causing progressive neurological deterioration in these patients.

Following traumatic SCI, the clinical entity of delayed neurological worsening up to many years post-injury is well established [14, 24, 27, 32, 34]. The condition is named post-traumatic syringomyelia or preferably progressive post-traumatic myelopathy (PPM). In accordance with previous findings, surgical untethering was safe in the present study and resulted in improved clinical outcome as evaluated by the JOACMEQ and EQ-5D assessment tools [14, 24, 27, 32]. However, re-tethering occurred in 25% of patients, suggesting a continuous process leading to recurrent intradural scar formation.

To date, the pathophysiology of PPM has not been fully clarified [24]. Hence, we assessed 3 biomarkers, namely GFAP, p-NFH, and UCH-L1, each linked to different cell origin and pathophysiological mechanisms that could help us appreciate the etiology and underlying processes predisposing to spinal cord tethering.

The intermediate filament cytoskeleton protein GFAP is an astroglial biomarker of CNS injury which is found in the astroglial skeleton of both white matter and gray matter with a suggested serum half-life of < 48 h [30]. Following thoracoabdominal aortic aneurysm surgery, CSF GFAP levels were > 500 times higher in patients who suffered ischemic spinal cord injury compared with controls [3]. Recently, in acute SCI patients, the GFAP levels in blood were higher in patients with motor complete injuries than in those with motor incomplete SCI, suggesting a correlation with the degree of cord injury [1, 19, 30, 42]. In our study, we did not observe a clear correlation between GFAP levels and the severity of the clinical situation or the time since the initial injury. This may be partially explained by the fact that spinal cord tethering, and its associated neurological exacerbation, is typically a slow process continuing over many years with large individual variability. Nonetheless, the increased GFAP levels in CSF suggest an active process presumably from glial scar formation that can represent a therapeutic target.

We also hypothesized that progressive white matter pathology could be associated with neurological deterioration in chronic SCI. We therefore evaluated pNF-H, a main component of the axonal cytoskeleton that has previously been associated with acute worsening of cervical spondylotic myelopathy [49], and injury severity in acute SCI [48]. pNF-H was substantially increased in CSF of patients with spinal cord tethering compared with controls, thus suggesting ongoing axonal degeneration in this population and supporting its use as a potential biomarker for chronic SCI.

UCH-L1 is a deubiquitinating enzyme present primarily in neurons [25]. It is a promising biomarker for many CNS disorders, including traumatic injuries [17, 38, 51]. It was also increased in a rat model of acute SCI [54], although has not thoroughly been evaluated in human SCI. In the present report, it was not elevated in chronic SCI patients, although a slight trend toward increased levels was discerned. Many factors might contribute to the discordance of these results including a subtle ongoing injury process which do not result in markedly increased levels of UCH-L1, as well as different biomarker kinetics and dynamics [8]. Taken together, these biomarker observations argue that spinal cord tethering is predominately characterized by glial and axonal involvement.

We observed a > 50-fold interindividual concentration differences in biomarker levels, particularly in plasma. Moreover, CSF levels were ~ 10-fold higher than in plasma and no correlation between the two compartments was observed. Significant differences among the groups were observed only in the CSF samples. Nonetheless, together, these data suggest that sampling of CSF is more sensitive to detect changes and altered biomarker profiles in chronic SCI patients than of plasma. These findings are in accordance with previous studies in other neurological diseases [5] and might be caused by many factors, including the blood-spinal cord barrier preventing free passage of biomarkers into the circulation, the use of research-grade assay, and the presence in blood of heterophilic antibodies, which may react with the immunochemical tests giving falsely low results. Nonetheless, together, these data suggest that sampling of CSF is more sensitive to detect changes and altered biomarker profiles in chronic SCI patients than of plasma.

Mounting evidence indicates that biomarkers become abnormal in a temporally ordered manner reflecting distinct contributing pathophysiological mechanisms. Following experimental and clinical traumatic brain injury, UCH-L1 and GFAP concentrations increase in biological fluids in the superacute (UCH-L1) and acute (UCH-L1, GFAP) phase and then, depending on injury severity, they normalize over 3–4 days unless secondary events occur [6, 41, 50, 57]. On the other hand, pNF-H is considered a subacute biomarker of axonal injury that rises in later stages [23, 46, 55]. In acute SCI, there is limited information on biomarker kinetics, although a similar trend has been reported [29–31].

In our patient cohort, the pattern of altered CSF pNF-H concentrations linked with no appreciable UCH-L1 changes implies that the neuronal/axonal injury is an earlier event which retains ongoing white matter degeneration. On the other hand, the observed increased GFAP concentrations in CSF, but not in blood, are consistent with continuing glial remodeling rather than a potential ensuing surgical injury, owing to the fact that the incision and surgical approach using microinstruments is mainly performed within dense scar tissue with minimal damage and bleeding [20]. In addition, CSF samples were taken immediately at dura opening, a time period likely insufficient to alter biomarker levels in our samples.

In the current cohort, GFAP levels but not UCH-L1 were significantly higher in tethered cord patients compared with radiculopathy. Since the surgical approach from skin to dural opening and CSF release did not exceed 1 h, UCH-L1 levels should arguably be even higher as a result of the surgical trauma indicative of a superacute phase. However, such results were not observed in our study sample. It must be also stressed that this is the first study to evaluate these biomarkers in patients operated for tethered cord, and therefore there are no previous results to compare with.

We used perioperative CSF sampling to, obtain CSF from the site of maximal injury, and avoided lumbar sampling since free CSF flow beyond the tethered area was uncertain. For comparison with the radiculopathy controls, CSF samples via a lumbar puncture would be preferred. However, this should be avoided in most tethered cord patients, due to multiple adhesions and stagnant CSF. Similarly, to obtain CSF via an ultrasound-guided tap is not applicable and, in particular, not safe due to the fact that spinal cord is commonly adhered to the dura. Moreover, it may be difficult to obtain a clear image by ultrasound when used on dense scar tissue. To percutaneously insert a needle through the dura at the level of the spinal cord, with the cord adherent to the dura and/or in patients with retained neurological function, should be regarded unsafe and unethical when used for scientific purposes.

One key aim of surgical untethering is to restore CSF flow by untethering the cord [14, 24, 32]. To mimic the surgical approach and in an attempt to evaluate whether the surgical trauma per se influenced the biomarker levels, we also included a small control cohort with intradural tumors not involving the spinal cord. It should be mentioned, however, that biomarker secretion from intradural tumors is not known and compression of cauda equina nerve roots may have influenced the biomarker results. These uncertainties were the main rationale for adding a radiculopathy control group. Thus, radiculopathy patients without spinal cord compression were also included as controls. Finally, future studies are needed to evaluate whether there are temporal alterations of biomarker abnormalities and their relationship with pathological stage, appearance of clinical symptoms, and disease progression.

The decision to proceed to this rather lengthy and potentially risky neurosurgical procedure depends on a combination of patient factors, degree of clinical deterioration, and to some extent the radiological findings. Currently, the indications for surgery may vary among centers due to the lack of objective measures. Biomarkers indicating glial scar formation and/or neuronal degeneration could, in combination with the degree of clinical neurological deterioration, provide more objective factors in determining the surgical indication. However, at present, it is premature to suggest their use as biomarkers in clinical practice, awaiting results from larger patient cohorts.

Our study has several limitations. The patient cohort is rather small, in view of the scarcity of the used surgical procedure. Therefore, the prognostic value of biomarker levels on outcome could not be determined. In addition, the study sample was insufficient to enable a multivariate analysis of the biomarker levels. For technical reasons at time of analysis, some results for plasma GFAP could not be obtained in a subset of patients. This could have influenced the statistical results, although in view of the limited changes overall in plasma compared with CSF, it is unlikely that it altered the main finding that biomarker changes in CSF were more robust than in plasma. The exact contribution of the surgical approach from skin incision to dural opening to CSF levels cannot be assessed although it is unlikely that it contributed significantly to the observed biomarker levels. Similarly, the degree of glial scar formation and neuronal degeneration could not be objectively evaluated. Importantly, biomarker results from patients who underwent first surgery and reoperation were similar. The biomarkers used in this cohort have previously been investigated in acute spinal cord injury, although not in chronic SCI patients and the normal levels in chronic spinal cord injury without tethering are unknown. Thus, this study is the first to evaluate serum and CSF biomarkers in patients with chronic spinal cord injury and intradural tethering of the cord. Lastly, the follow-up period was relatively short. However, recent work from our group showed that long-term outcome following untethering is favorable [24]. Therefore, we have no reason to believe that the outcome would change substantially between 3 and 12 months. Moreover, the primary aim of surgical untethering is to arrest ongoing clinical deterioration, not improvement per se.

## Conclusions

In this report, we evaluated CSF and plasma biomarkers in patients with spinal cord tethering and intradural scar formation. The patient cohort was heterogeneous and consisted of patients with previous trauma, surgery, infection, or hemorrhage. We were interested in unveiling mechanisms causing the delayed neurological deterioration in these patients using a multimarker panel. We showed that compared to controls, GFAP and pNF-H levels in CSF but not in plasma were higher in patients surgically treated for tethered spinal cord, indicating that CSF is superior to blood biomarker sampling in this population. The present results also suggest that biomarkers may provide insight, as observed in other acute CNS disorders, into the pathophysiology of a progressive axonal injury and glial scar formation plausibly contributing to spinal cord dysfunction and progressive neurological deterioration observed in a subset of chronic SCI patients. However, this assumption should be validated in a larger cohort of SCI patients, preferably with histological verification, when possible.
